# Supplementary material for: Predicting soil thickness on soil mantled hillslopes
Source: Nat Commun. 2018 Aug 20;9:3329. doi: 10.1038/s41467-018-05743-y (PMC6102209; doi:10.1038/s41467-018-05743-y)
Supplement: Supplementary file 1 — Supplementary Information [file 41467_2018_5743_MOESM1_ESM.pdf]

Supplementary Information

Predicting soil thickness on soil mantled hillslopes

Patton *et al.*

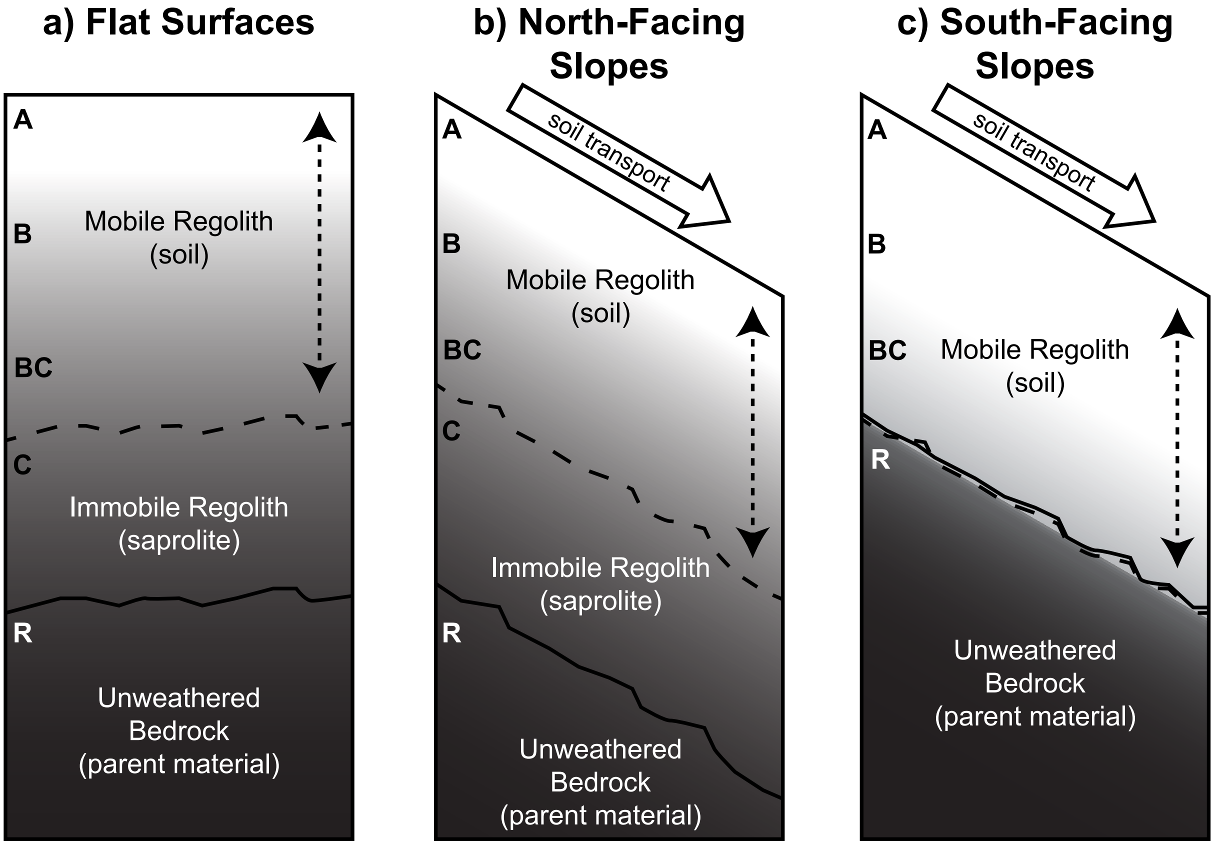

Supplementary Figure 1 Thickness of mobile regolith Conceptual diagram of the contact (thick black dashed line) between thickness of mobile regolith (TMR) and immobile regolith for both (a) flat, (b) north- and (c) south-facing hillslope surfaces. TMR differs from total regolith or total soil depth by not including the immobile regolith. Master horizons are indicated by mineral soil (A horizon), subsoil (B horizon), partially weathered bedrock (C horizon), and regolith (R). The base of the transitional subsoil to partially weathered bedrock (BC horizon) is typically the base of the mobile regolith.

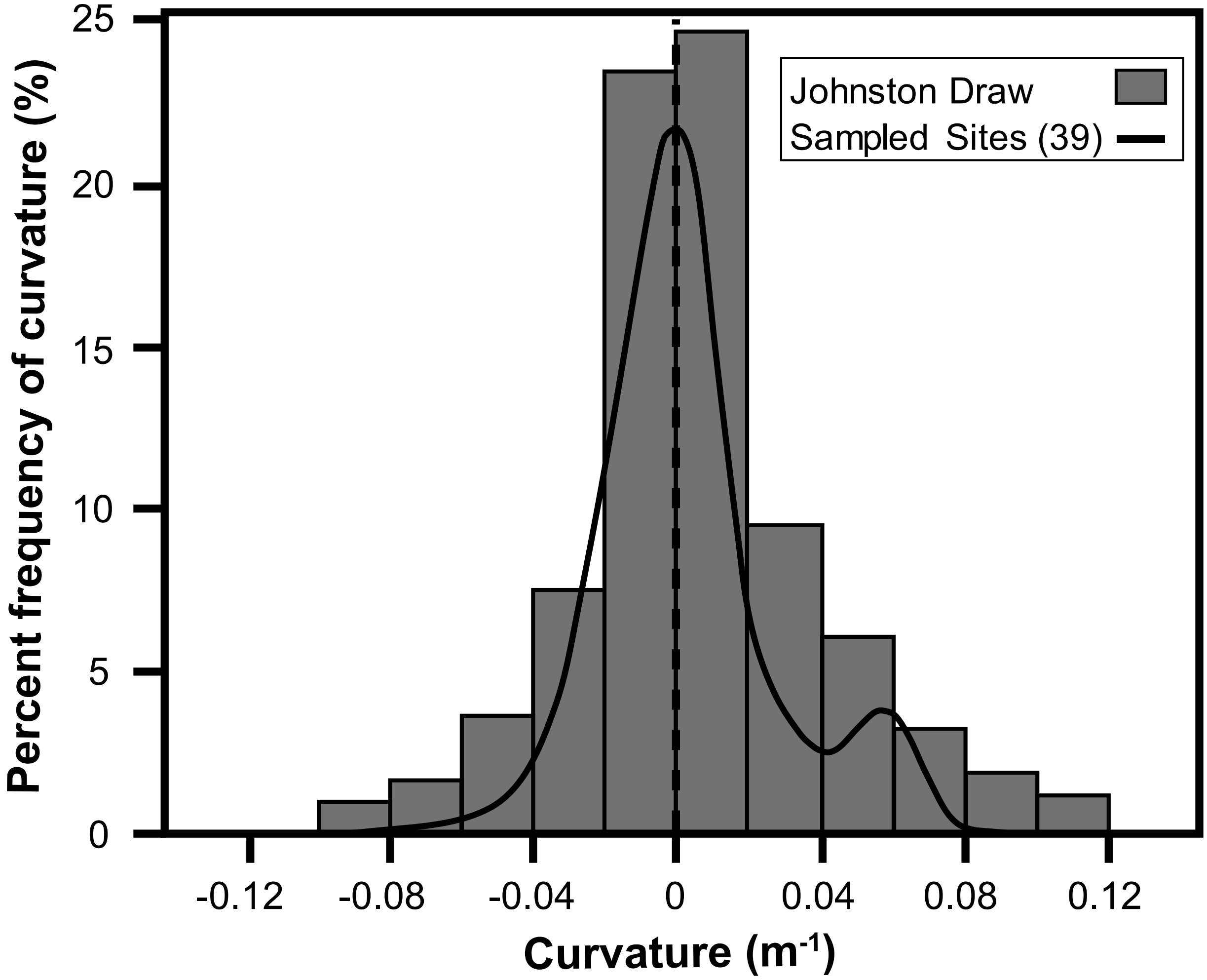

Supplementary Figure 2 Curvature distributions Both histograms for sampled sites (black line) (N=39) and the entire Johnston Draw (gray bars) show an approximately normal distribution with the highest frequency at 0 m^{-1}. However, sampled sites in high curvature areas, 0.04 m^{-1} through 0.08 m^{-1}, are overrepresented because of slight oversampling in one stratum of the stratified random site selection.

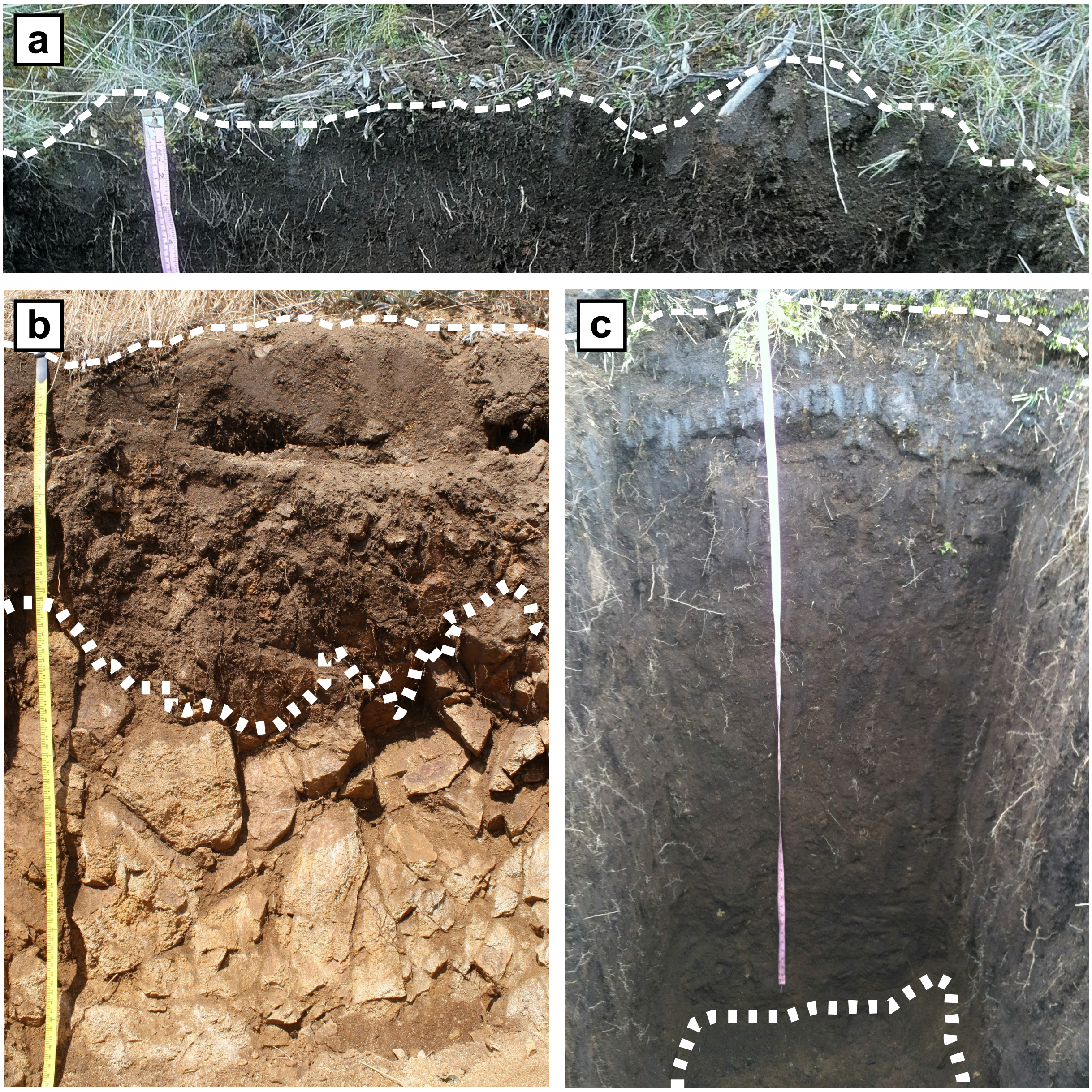

Supplementary Figure 3 Surface roughness and mobile-immobile regolith boundaries Photograph of typical surface roughness (dashed white line) within Johnston Draw catchment at pit JDT 3d (IGSN IERCO002U). Surface elevations vary by approximately ± 0.05 m (a). Photograph of south-facing soil pit, JDT 2g (IGSN IERCO002M), showing a clear, irregular mobile regolith-weathered bedrock boundary at 0.20 ± 0.05 m (thick-dashed white line) [photo source: A. Rozin] (b). Photograph of north-facing soil pit, JDT 3e (IGSN IERCO002V), showing a diffuse mobile regolith-weathered bedrock boundary at 2.13 ± 0.20 m (c).

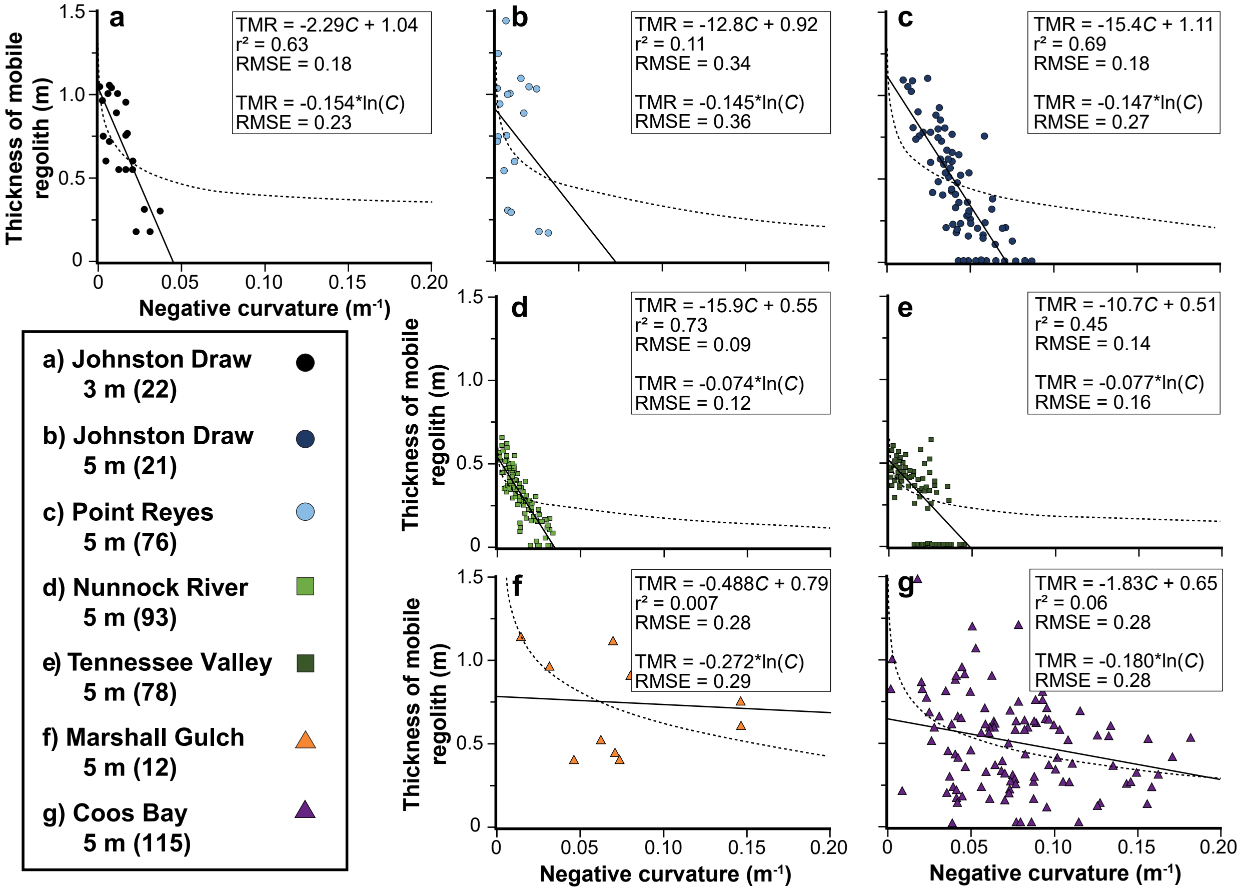

Supplementary Figure 4 Evaluation of linear vs natural logarithm functions for the thickness of mobile regolith (TMR) and curvature (*C*) Cross-site evaluation of six catchments in which the linear TMR-curvature function (solid line) is compared to the natural logarithm TMR-curvature function (dashed line) as described in theoretical models. A 5-m digital elevation model (DEM) is used with the exception of (a) in which a 3-m DEM was used. We note that we retained the negative curvature convention[2,3] in this analysis to be able to perform the natural logarithm curve fit. We observed that the linear model outperforms the natural logarithm in all cases except (f) and (g) where both models perform poorly.

Supplementary Table 1 Horizontal and vertical uncertainty in Light and Ranging (LiDAR) data

Catchment	Horizontal Uncertainty (m)	Vertical Uncertainty (m)	TMR Uncertainty (m)	Curvature Uncertainty at r = 0 (1/m)
Tennessee Valley	n/a	n/a	0.061	0.018
Coos Bay	0.4	0.073	0.071	0.013
Point Reyes	n/a	0.0925	0.141	0.017
Marshall Gulch	1	0.37	0.082	0.066
Johnston Draw	n/a	0.034	0.130	0.006
Babbington Creek	n/a	0.034	0.059	0.006
Gordon Gulch	0.11	0.175	0.040	0.003
Nunnock River	n/a	n/a	0.103	0.018
Reynolds Mountain	n/a	0.034	0.110	0.006

Horizontal and vertical uncertainty are obtained through metadata provided on OpenTopography.org, n/a represents where data was not provided. Curvature uncertainty as measured by standard error was calculated by the Method of Moments assuming correlation between uncertainty of neighbor and center cell points are 0 (r = 0). When vertical uncertainty is not provided, we assume an uncertainty of 0.1 m.
